# The *Tofu* Mutation Restores Female Fertility to *Drosophila* with a Null *BEAF* Mutation

**DOI:** 10.3390/genes17030328

**Published:** 2026-03-17

**Authors:** J. Keller McKowen, Maheshi Dassanayake, Craig M. Hart

**Affiliations:** Department of Biological Sciences, Louisiana State University, Baton Rouge, LA 70803, USA

**Keywords:** *Drosophila melanogaster*, female fertility, meiotic recombination, insulator proteins, Polycomb response element, single-nucleotide polymorphisms

## Abstract

**Background**: Compensatory mutations offer clues in deciphering the role of a particular protein in cellular processes. Here, we investigate an unknown compensatory mutation, present in the *BEAF^NP6377^* fly line, that provides sufficient rescue of the defective ovary phenotype caused by null *BEAF* alleles to allow the maintenance of fly stocks lacking the chromatin domain insulator proteins Boundary Element-Associated Factors BEAF-32A and BEAF-32B. We call this dominant mutation *Tofu*. **Methods**: We employ both classical genetics and genomic sequencing to attempt to identify the mutation. **Results**: We find evidence that points to a mutation in a predicted Polycomb response element (PRE) upstream of the *ribbon* transcription factor gene. This may lead to aberrant *rib* expression, which is otherwise not expressed in adult ovaries. BEAF and Rib colocalize to a set of promoters, suggesting overlap in gene regulation. **Conclusions**: *Tofu* could be a PRE mutation leading to the aberrant activation of *rib* in the ovaries. This could allow Rib to compensate for a lack of BEAF to activate one or more coregulated genes necessary for egg production in flies.

## 1. Introduction

Boundary Element-Associated Factor of 32 kDa (BEAF) is an insulator-binding protein of *Drosophila melanogaster* that mainly binds in promoter regions of housekeeping genes [1,2,3]. The *BEAF* gene is located on the second chromosome. It uses alternative promoters to create two isoforms: BEAF-32A and BEAF-32B. Each isoform has a unique N-terminal BED finger DNA-binding domain of 81 (32A) or 80 (32B) amino acids [4]. The C-terminal 202 amino acids are common to both isoforms [5]. This contains a C-terminal BESS (BEAF, Su(var)3-7, Stonewall) domain that mediates BEAF-BEAF interactions, with a leucine zipper immediately adjacent to the BESS domain that strengthens these interactions [6,7]. Previously, our lab generated and characterized a null allele of *BEAF* by eliminating the ATG start codons of both the 32A- and 32B-specific exons and introducing two tandem stop codons into the shared exon, *BEAF^AB-KO^* (*ABKO*). Flies homozygous for the *ABKO* allele are sickly, display a mild rough eye phenotype, and have defects in oogenesis, while males remain fertile. A 32B transgene rescues these phenotypes, while a 32A transgene does not. The ovaries of *ABKO* flies have malformed egg chambers, displaying variable phenotypes that are most apparent at stage eight of oogenesis. As a result, very few mature oocytes are produced. Because of the sharp loss of fecundity, *ABKO* homozygotes cannot be maintained as a stock [8].

**Figure 1:**
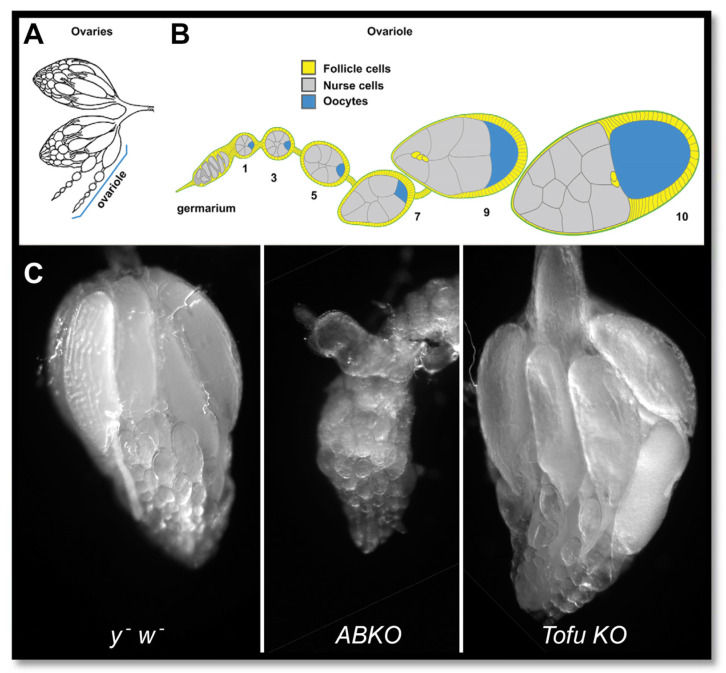
Rescue of the *BEAF^AB-KO^* ovary phenotype by *Tofu*. (**A**) Line drawing of typical fly ovary morphology. Ovaries are composed of several ovarioles, which each produce oocytes. (**B**) Oocyte development proceeds from the stem cells at the tip of the germarium to the egg chambers, which can be classified as stages by phenotype as numbered. Stage 14 is the mature egg. (**C**) Ovary phenotypes of 5- to 7-day-old, mated *y^-^ w^-^*, *ABKO*, and *Tofu KO* flies. *Tofu KO* ovaries have mature eggs but are atypical. [A and B reproduced with permission from [12].

A separate null allele, *BEAF^NP6377^* (*NP6377*), was generated by the insertion of a GAL4 enhancer trap element containing a mini-*white* marker gene, *P(GawB)*, near the 5′ end of the exon shared by the 32A and 32B isoforms [9]. Thus, in contrast to the *ABKO* allele, the *NP6377* allele is marked by *w*. This *NP6377* line was used in a study that attributed the striking phenotypes of neoplastic growth and recessive larval lethality to the loss of BEAF [10], which were not observed in our *ABKO* mutant line. To investigate, we obtained the balanced *NP6377* line. We were not able to rescue the phenotypes with two different *BEAF* transgenes that were able to rescue *ABKO* flies [11]. The balanced *NP6377* and *ABKO* lines were also crossed in a complementation test, since they were independently generated and so should not have the same second site mutations. The test showed the rescue of the recessive larval lethal and neoplastic growth phenotypes, suggesting that they were not a consequence of the loss of BEAF. Surprisingly, *NP6377/ABKO* flies were viable, although their ovaries were not completely normal (Figure 1). The defect in fertility caused by the lack of BEAF was suppressed by a dominant mutation from the balanced *NP6377* line [11]. We named this mutation *Tofu* because it allows fly stocks to be maintained without BEAF. By meiotic recombination in the *NP6377/ABKO* line, we generated *ABKO/ABKO* (*Tofu KO*) and *NP6377/NP6377* (*Tofu NP)* lines. The recombinant lines lacked BEAF and any recessive lethal mutations, but they retained the dominant enhancer of fertility mutation and appear to have nearly wild-type fertility. Here, we describe attempts to locate *Tofu* using two methods: classical genetic mapping via meiotic recombination and high-throughput genomic DNA sequencing.

## 2. Materials and Methods

### 2.1. Fly Stocks

Flies were maintained on cornmeal, yeast, and sugar medium with Tegosept (per liter H_2_O: 60 g cornmeal; 36 g yeast; 42 g sucrose; 7 g agar; 4 g Tegosept). *BEAF^NP6377^/CyO GFP* fly stocks were kindly provided by Victor Corces (RRID: DGGR 05221). *BEAF^AB-KO^/CyO GFP* fly stocks (*CyO GFP* from RRID BDSC 5194) were previously generated by our lab [8]. *Tofu BEAF^N6377P^* and *Tofu BEAF^AB-KO^* lines were created by meiotic recombination from a *BEAF^AB-KO^*/*BEAF^NP6377^* stock [11]. Baylor *P* mapping kit lines (2L: 27E6, 30C1, 33A2, 34B4, 35D2, 36E3, 37B13; 2R: 41D4, 43E11, 47A11, 49E1, 53D4, 55F8, 57E9), *P[w^+^]* at 56C6 (RRID: BDSC 18385) and 57B16 (RRID: BDSC 16087), and balancer chromosome lines were obtained from the Bloomington *Drosophila* Stock Center.

### 2.2. Fly Genotyping

To detect the *ABKO* allele, we designed a genotyping primer set consisting of three primers. The BEAF-wt-Bam-5 has 5′ homology to the *Bam*HI site in the WT *BEAF* allele. The BEAF-mut-5 primer has 5′ homology with the stop codon mutation introduced into the *Bam*HI site of the *ABKO* allele [8]. The 3′ BEAF-stop+89-3 primer was used with both of these primers to generate a roughly 500 bp product from the corresponding *BEAF* allele. Genotyping was performed using Phire tissue direct master mix (Thermo-Fisher F-170, Waltham, MA, USA). Briefly, one headless fly was mashed in 20 μL dilution buffer plus 0.5 μL DNARelease additive to release DNA, according to the manufacturer’s protocol. Then, 1 μL was added to 9 μL H_2_O plus 10 μL 2x Phire tissue direct master mix and heated at 95 °C for 5 min, followed by 35 cycles of 95 °C 10 s denaturation, 60 °C 10 s annealing, and 72 °C 30 s elongation with a 60 s final elongation.

BEAF-wt-Bam-5: GACATCATATACAGCGAGGATCC

BEAF-mut-Bam-5: AAGGACATCATATACAGCGAGTAATG

BEAF-stop+89-3: TTACGACACGCTGATTTGCC

### 2.3. Genomic DNA Preparation and Analysis

Fifty wandering third-instar larvae from the y^-^ w^-^, *ABKO/CyO-GFP*, *NP6677/CyO-GFP*, *Tofu KO*, and *Tofu NP* lines were used for DNA isolation. Larvae homozygous for *BEAF* knockout alleles were selected from the *ABKO/CyO-GFP* and *NP6677/CyO-GFP* lines by lack of GFP expression. Larvae were homogenized in 500 µL of Buffer A (10 mM Tris-Cl pH 7.5, 60 mM NaCl, 10 mM EDTA, 150 µM spermine, 150 µM spermidine, 200 µg/mL Proteinase K). Then, 500 µL of Buffer B (200 mM Tris-Cl (pH 7.5), 30 mM EDTA, 2% SDS, 200 µg/mL Proteinase K) was added, and the samples were incubated for one hour at 37 °C. Samples were purified by phenol extraction, followed by phenol–chloroform–isoamyl alcohol (25:24:1) extraction, and then by chloroform extraction. Samples were ethanol-precipitated and dissolved in 10 mM Tris-Cl pH 7.5.

#### 2.3.1. Illumina Sequencing

Genomic DNA was submitted to the Roy J. Carver Biotechnology Center at the University of Illinois at Urbana. Libraries were prepared from 300 bp to 500 bp fragments for each sample, followed by the paired-end sequencing of 150 bp from each end using an Illumina HiSeq2500 instrument.

#### 2.3.2. Data Analysis

Raw reads were obtained as paired fastq files (deposited at NCBI BioProject accession number PRJNA1069327). Read quality was analyzed using FASTQC v0.11.3 [13]. Reads were aligned to the dmel r6.09 genome [14] using bowtie2 v2.2.5. Samtools v1.2 was used to assess alignment quality and compress sam alignments into bam format. Variant calling was performed using the GATK toolkit v3.4 [15,16,17]. Variant call files (VCF) were compared using bedTools v2.23.0 [18]. Protein coding changes were called with SnpEff v4.1 [19]. SNP density was visualized as bigwig files using 2000 bp windows. File conversion was performed using Unix commands, Kent utilities v302 [20], bedTools, and deepTools v2.0.1 [21]. Data was displayed using IGV v2.3.57 [22].

## 3. Results

### 3.1. Tofu Is on Chromosome 2

**Figure 2:**
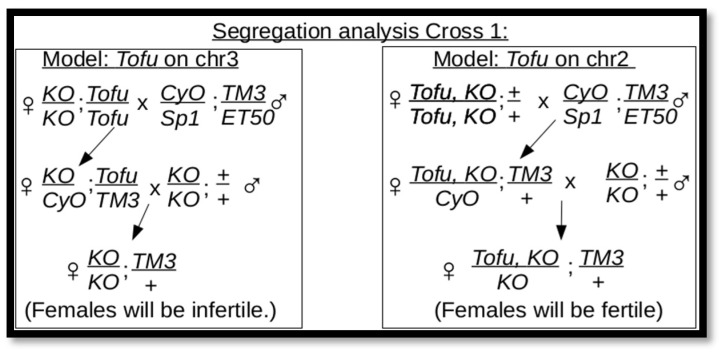
Diagram of a segregation cross for chromosomes 2 and 3. The mating strategy takes advantage of the fertility of *ABKO* males and is divided into two models of expected results. A female fertility rate of 53% was found, rather than the expected 0% or 100%.

The discovery of the *Tofu* allele indicated that it was on the same chromosome as *BEAF*—chromosome 2 [11]. To check this, we performed a chromosome segregation analysis. In the first strategy (Figure 2), *Tofu KO* flies were mated with a *CyO/Wg^Sp-1^;TM3/Scm^ET50^* double balancer line. We assayed 200 females from the second cross (one female with three males per vial) and found a fertility rate of 53%. This indicates that *Tofu* is not on chromosome 3, but it does not clearly support an association with chromosome 2. *Tofu* could be on chromosome 2 if it was at least partially heterozygous in the *Tofu KO* stock and/or some of the single-pair mating vials were scored as infertile because the vials were not healthy. The incomplete rescue of ovary function (Figure 1) and other possible unknown fitness issues could also contribute. *Tofu* could also be on chromosome X or 4.

**Figure 3:**
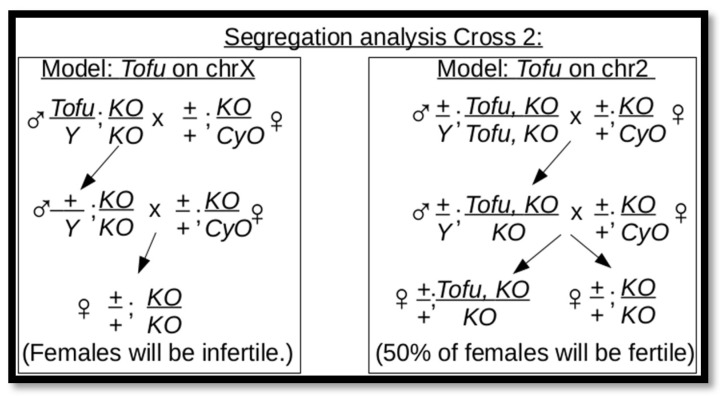
Diagram of a segregation cross for chromosomes X and 2. The mating strategy is divided into two models of expected results. A female fertility rate of 52% was found.

In the second strategy (Figure 3), males of the *Tofu KO* line were mated to *ABKO/CyO* virgin females. We assayed 200 female progeny from the second cross and found a fertility rate of 52%. This indicates that *Tofu* is not on chromosome X and suggests that it is associated with chromosome 2. This strategy does not exclude the possibility that *Tofu* is on chromosome 4, although, given the small size and low number of genes, this is unlikely. Moreover, single *Tofu KO*/*CyO* and *Tofu NP*/*CyO* flies could be crossed to *CyO*/*Sp1* for several generations and retain the ability to produce *Tofu KO* and *Tofu NP* homozygous lines. If *Tofu* were on chromosome 4, there would be a 50% chance of losing the ability to produce homozygous lines with each cross after the first cross. By eliminating the other chromosomes, this indicates that *Tofu* is on the same chromosome as the *BEAF* alleles.

### 3.2. Mapping by Meiotic Recombination

**Figure 4:**
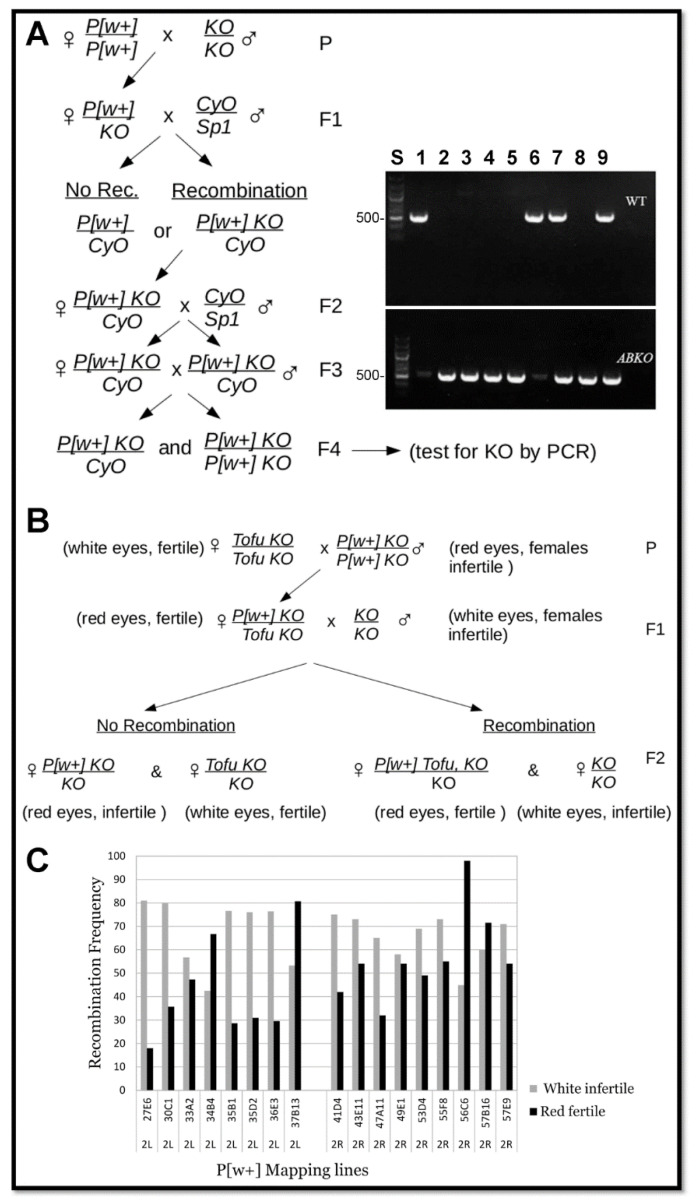
Meiotic recombination mapping strategy. (**A**) Strategy for recombining *P[w^+^]* onto the *ABKO* chromosome and PCR detection of recombination for 27E6 (535 bp PCR product for *wt* and *ABKO*). Agarose gel lanes—S: standard (500 bp marker is indicated); 1: *wt* fly; 2–8: red-eyed recombination candidate flies (homozygous males except lane 7, which is a female with *CyO*); lane 9: *ABKO*/*CyO* fly. PCR primers used for the *wt* allele (upper tier) or *ABKO* allele (lower tier). Recombination occurred for all candidates except lane 6 (*wt BEAF* in lane 7 is from the *CyO* chromosome). (**B**) Mapping strategy. (**C**) Flies from the mapping strategy were segregated based on eye color and individual females were scored for fertility (White: *n* = 100; Red: *n* = 100).

To narrow down the region that contained *Tofu*, we employed meiotic recombination in females to map genetic linkage. Fly lines with chromosome 2 *w^+^* P element insertions at known cytological locations from the Baylor P mapping kit were used [23]. The *ABKO* allele was recombined onto the *P[w^+^]* chromosome and confirmed by PCR (Figure 4A). This chromosome was then used in the strategy shown in Figure 4B to obtain the recombination frequency of *Tofu* from the *Tofu ABKO* chromosome and onto the *P[w^+^] ABKO* chromosome, using the fertility of individual females as the phenotype. If *Tofu* recombined onto the *P[w^+^]* chromosome, females with red eyes should have been fertile and those with white eyes should have been infertile. For each *P[w^+^]* line, we scored 100 single-pair matings for females with red eyes and 100 for females with white eyes. The anticipated result was that the percentage of fertile females with red eyes would equal the percentage of infertile females with white eyes, and the percentage would be much lower than 50% if *P[w^+^]* and *Tofu* were cytologically close. These single-pair matings proved to be unreliable (Figure 4C), possibly because of unknown genetic interactions with genes from the *P[w^+^]* mapping lines or unhealthy environmental conditions in some single-pair mating vials.

**Figure 5:**
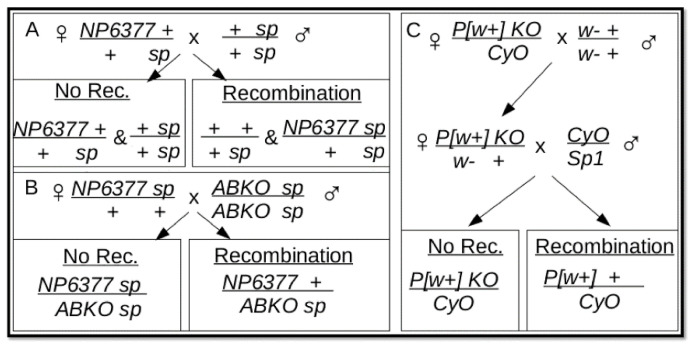
Meiotic recombination mapping strategy for *BEAF* alleles at 51C2 relative to *speck* at 60B12-60C1 and *P[w^+^]* at 49E1 and 53D4. (**A**) Strategy for detecting recombination of *speck* (*sp*) onto the *NP6377* chromosome. A recombination rate of 49% was found (*n* = 305). The *w^+^* transgene in the *NP6377 BEAF* allele was used for identification. (**B**) Strategy for detecting recombination of *sp* from the *NP6377* chromosome. A recombination rate of 56% was found (*n* = 111). (**C**) Strategy for detecting recombination of *P[w^+^]* from the *ABKO* chromosome. Presence or absence of *ABKO* was detected by PCR of flies with red eyes, as in Figure 4A. A recombination rate of 7.6% was found for 49E1 (*n* = 79) and 11.3% for 53D4 (*n* = 71). Flies in all crosses had a *w^-^* chromosome X.

We tested our ability to perform meiotic mapping using two strategies. This also allowed us to determine the recombination frequencies of cytological locations relative to the *BEAF* gene near the center of chromosome arm 2R at 51C2. First, we used the recessive marker mutation *speck* (*sp*), which is in the telomere-proximal 60B12-60C1 region of 2R (~9.3 Mb from *BEAF*). This mutation was present in our *ABKO* and *Tofu KO* lines but not in the *NP* lines. Using the strategies shown in Figure 5A,B, we found that *sp* recombined onto the chromosome with *NP* with a frequency of 49% (*n* = 305) and off again with a frequency of 56% (*n* = 111). Second, we used P mapping lines with *ABKO* recombined onto the two chromosomes with *P[w^+^]* inserted nearest to *BEAF*, 49E1 and 53D4 (~1.8 Mb and 1.5 Mb from *BEAF*, respectively). Using the strategy shown in Figure 5C and PCR to determine if the *ABKO* allele was present, we found that *ABKO* separated from the 49E1 *P[w^+^]* with a frequency of 7.6% (*n* = 79) and from 53D4 *P[w^+^]* with a frequency of 11.3% (*n* = 71). The results demonstrated our ability to identify linked regions, confirming that the failure of our fertility assay was due to problems in accurately scoring fertility. Complex traits such as female fertility could be particularly difficult to score. Contributing factors could include sensitivity to disruption because of incomplete rescue by *Tofu* or pleiotropic effects on fly fitness in homozygous *ABKO* flies [8] that might not be rescued by *Tofu*, combined with single-pair matings where vials are more prone to developing unhealthy environmental conditions.

### 3.3. Genomic Sequencing

In another approach to identifying *Tofu* candidates, we used genomic sequencing to compare mutations among fly lines. Genomic DNA was isolated from homozygous larvae of five lines: *y^-^* *w^-^*, *ABKO*, *NP6377*, and the two lines generated from recombination—*Tofu NP* and *Tofu KO*. The *Tofu* mutation was in *NP6377*, *Tofu NP,* and *Tofu KO* but not *ABKO* and *y^-^ w^-^*. Illumina sequencing was used to generate paired-end reads of 150 bp per end from 300–500 bp DNA fragments [24]. Sequence reads were aligned to the dm6 genome release for *D. melanogaster* using bowtie2 after filtering for quality and trimming adapters, resulting in genome coverage of over 40 reads per base (Table 1). Paired-end sequencing improves the alignment accuracy, and an excessive distance between read pairs can indicate an insertion mutation relative to the reference genome [25,26].

The aligned genomes were used to call variants relative to the dm6 reference genome by using a suite of programs in the genome analysis toolkit, GATK [15,16,17]. The resulting variant call files (VCFs) for each fly line detail the location and sequence change from the reference genome for single-nucleotide polymorphisms (SNPs) and extra bases or missing bases (insertions or deletions, lumped together as INDELs) (Supplementary Files S1–S5). For simplicity of discussion, we will use SNPs to refer to both SNPs and INDELs. The VCFs for each of the five lines had over 700,000 SNPs relative to the reference genome (Table 2). After cross-comparing, we found 61,693 SNPs that fit the pattern for *Tofu* among the lines sequenced (Supplementary File S6). Of these, 1203 SNPs were localized to chromosome 2—a small number compared to other chromosomes. We reasoned that this was due to recombination-mediated shuffling and selection between the original *ABKO* and *NP6377* chromosomes to place *Tofu* onto the *ABKO* chromosome while removing recessive lethal mutations from the *NP6377* chromosome.

We used SnpEff to predict the effects of the chromosome 2 SNPs (Supplementary File S7, summarized in Supplementary Table S1). Focusing on protein synthesis, we found that 35 of the SNPs would cause missense, frameshift, or splicing mutations in 17 protein-coding genes. Only two of these genes emerged as likely candidates. One, *18 wheeler* (*18w*), encodes a Toll-like receptor family member and plays a role in various processes, including ovarian follicle cell migration [27]. However, *18w* has a conservative Ala1357Val mutation near its C-terminus that might not affect function. The other, *hu li tai shao* (*hts*), encodes an adducin homolog that is associated with oocyte fusomes and ring canals and is essential for fertility [28,29]. However, the three missense mutations in *hts* (Lys1760Glu, Ile1764Met, Thr1773Asn) affect only 1 of 16 splice variants (*hts-RP*), and this isoform has not been found in the ovaries ([30] and FlyBase JBrowse RNA-seq data). Therefore, we focused on the possibility that *Tofu* was not a gain-of-function protein-coding mutation.

**Figure 6:**
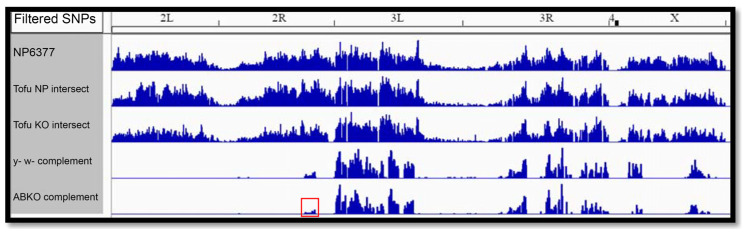
IGV visualization of SNP density. The SNPs are filtered from top (unfiltered) down. The top 3 genomes have *Tofu*; the bottom 2 do not. The bottom row shows candidate SNPs for *Tofu* with the 2R SNP island boxed in red.

We re-examined the *Tofu* candidate variants by plotting them using the Integrative Genomics Viewer (IGV) [22]. Meiotic recombination between the original *NP6377* and *ABKO* chromosomes generated the *Tofu NP* and *Tofu KO* fly lines by removing one or more recessive lethal mutations from the *NP6377* chromosome and transferring *Tofu* to the *ABKO* chromosome. We reasoned that this process would also transfer linked variants near *Tofu*, so the *Tofu* mutation should lie in a variant-dense region. While there are several variant-dense regions, only one is on chromosome 2 (Figure 6). This region on 2R contains 954 of the 1203 candidate SNPs on the second chromosome, including 32 of the 35 SNPs that changed protein-coding sequences. We refer to this region as the “SNP island”.

The SNP island corresponds to cytological regions 55F8-57C3 (dm6 chr2R:18,868,928–21,135,871, nearly 2.3 Mb). The original P-element fly lines that we used were outside of this region, so we obtained *P[w^+^]* insertion lines within the SNP island at 56C6 and 57B16. The recombination rates were higher than expected if *Tofu* is in this region (Figure 4). Because we suspect that *Tofu* is in this region, these results reinforce our reservations about the reliability of the fertility assay.

**Figure 7:**
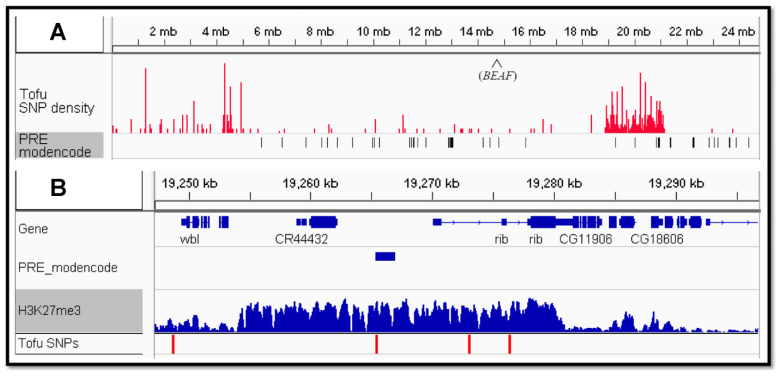
An SNP in a predicted PRE upstream of the *rib* gene is a candidate for the *Tofu* mutation. (**A**) IGV screenshot of chromosome 2R showing SNP distribution of *Tofu* candidates (red) and predicted PREs (black). Note the SNP island centered around 20 Mb. (**B**) IGV screenshot of the candidate PRE upstream of the *rib* gene. Data for the histone H3K27me3 mark associated with Polycomb repression is from the ovaries (SRX177656). PRE_modencode data are available at FlyBase.org. PRE SNP: 2R:19,265,447 (AATGCTGCTGCTCGA[T to G]CCTGACAAGCGTGTG).

Rather than a mutation in protein-coding sequences, *Tofu* could be a cis-regulatory element (CRE) mutation. As a dominant mutation, *Tofu* would likely activate a gene in the ovaries by creating a new enhancer, altering the tissue specificity of an existing enhancer, or inactivating a repressor. The intersection of *Tofu* candidates with the REDfly database of CREs [31] found 287 SNPs distributed among 181 CREs (Supplementary Table S2). Of these, 247 SNPs and 143 CREs are in the SNP island. The quality of the CRE database is unclear—over half of the CREs with potential *Tofu* SNPs are over 2 kb, which is large for a regulatory element. In addition, a given CRE can alter target gene expression from great distances, and CRE promoter specificity is not well characterized for most genes, making it difficult to identify target genes. Lastly, it can be difficult to predict if a SNP will alter factor binding. Our initial inspection did not find any strong candidates. Thus, we decided to also look for mutations in Polycomb response elements (PREs). A PRE can facilitate the initiation and spreading of a histone H3K27me3 domain, a mark associated with the repression of genes located in the domain [32]. A PRE mutation could prevent the establishment of a repressive histone H3K27me3 domain, resulting in the increased transcription of genes normally in the domain. This would be consistent with the dominant *Tofu* mutation. A previous study predicted 537 PREs based on chromatin analysis [33]. We found only two second chromosome predicted PREs with candidate SNPs, and both are within the SNP island region of 2R (Figure 7A; Supplementary Table S3). One is upstream of the gene *Act57B*, while the other is upstream of the *ribbon* (*rib*) BTB/POZ domain transcription factor gene (Figure 7B). While other SNPs could be responsible for restoring female fertility, as described next, we believe that the PRE SNP upstream of *rib* is a strong candidate.

Our lab previously performed the co-immunoprecipitation of BEAF from *Drosophila* embryos and, by tandem mass spectrometry, identified around 100 co-immunoprecipitated factors with more than one peptide identified, a Benjamini–Hochberg-corrected *p*-value less than 0.10, and a ratio of experimental to control IP greater than 1.0 [34]. One of these was *rib* (three peptides, 1.8-fold enriched, 0.0390 *p*-value), making the SNP in the upstream PRE a candidate for *Tofu*. ChIP-seq data from the ovaries (SRX177656) shows that the PRE upstream of *rib* is in a 25 kb island of the histone H3K27me3 mark that is associated with Polycomb silencing [35], suggesting that *rib* is normally silenced in the ovaries. Consistent with this, we examined a single-cell atlas of gene expression in the *Drosophila* ovaries [36] and found that *rib* expression was not detectable in any adult ovarian cell types. On the other hand, *rib* is involved in embryonic gonad development [37]. It also has developmental roles in the Malpighian tubules, salivary glands, hindgut, tracheae, and head [38]. The PRE SNP could cause *rib* to be expressed in adult ovaries, where it could compensate for the loss of BEAF to rescue the fertility defect.

Additional considerations are consistent with the idea that the misexpression of *rib* in the ovaries could rescue the fertility defect caused by null *BEAF* mutations. ChIP-seq data from embryonic salivary glands identified 494 Rib-associated genes in the developing glands [39]. We previously generated a list of 3241 BEAF-associated genes from S2 cell ChIP-seq data [34,40] and found overlap with 30% (150) of the Rib-associated genes. A motif related to the BEAF-32B DNA-binding consensus motif, CGATA [3,5], represented around 15% of the occurrences of the top 10 motifs found in the Rib ChIP-seq peaks associated with the 494 genes [39]. A more recent study found that Rib is associated with 73 of 84 ribosomal protein genes that are active in developing salivary glands and increases the expression of these genes, at least partially accounting for the requirement for Rib for salivary gland development [41]. This work failed to detect sequence-specific DNA binding by Rib, suggesting that it is recruited to DNA by other proteins. Our analysis of S2 cell BEAF ChIP-seq data found that BEAF is associated with 61 of these ribosomal protein genes, with both Rib and BEAF found at 53 of these genes. We also demonstrated that BEAF could activate four tested ribosomal protein promoters (*RpS12*; *RpL10Ab*; *RpLP1*; *RpL26*) in a transient transfection assay [42,43]. BEAF could be one of several factors that helps to recruit Rib to promoters. Other regulators of ribosomal protein gene expression (TRF2, M1BP, DREF) can co-immunoprecipitate Rib, making them additional candidates [41]. If the SNP in the predicted PRE directly upstream of the *rib* promoter leads to *rib* activation in the adult ovaries, Rib could replace the function of BEAF at genes such as those encoding ribosomal proteins to largely restore ovary function.

## 4. Discussion

The fortuitous discovery of *Tofu* and finding that it is on chromosome 2, as is *BEAF*, led us to try to determine its location relative to *BEAF* by meiotic mapping. Our attempts at mapping were unsuccessful due to the difficulty in scoring fertility. Because the *BEAF^AB-KO^* and *BEAF^NP6377^* mutations were independently derived [8,9] but the *Tofu ABKO* and *Tofu NP* chromosomes were related by recombination, we decided to try using genomic sequencing to identify *Tofu*.

In retrospect, our genomic sequencing did not sample enough populations containing the *Tofu* mutation. Genome-wide association studies in humans use many genomes from individuals to identify SNPs associated with a trait. These studies include a minimum of 2000 individuals in each group, often exceeding 100,000 [44,45]. Our analysis was limited to five genomes of pooled populations, and this likely limited our ability to screen SNPs. However, similar pooled sequencing strategies have been employed successfully in *Drosophila* [46].

Because *Tofu* is a dominant mutation, we originally focused on SNPs that might cause a gain-of-function protein-coding change. The lack of strong candidates led us to consider cis-regulatory element mutations. We also realized that SNPs near *Tofu* would likely remain associated with *Tofu*, so we looked for chromosome 2 SNP islands in our data. There was one island, and it contained 80% of the chromosome 2 SNPs. This did not significantly narrow down the number of SNPs to consider, but it did localize a region that likely contained *Tofu*. CRE mutations and affected genes can be difficult to identify, but we considered a silencer element or repressive chromatin domain as likely candidates and took advantage of predicted PREs [33]. Only two predicted PREs in the SNP island contained SNPs, leading us to focus on a predicted PRE just upstream of the *rib* gene. The region around this PRE, including *rib*, is in a chromatin domain enriched with the repressive histone H3K27me3 modification in the ovaries [35], and *rib* expression was not detected in a single-cell atlas of ovary gene expression [36]. If *Tofu* is a PRE mutation that leads to the aberrant activation of *rib* in the ovaries, it could allow Rib to compensate for a lack of BEAF to activate one or more coregulated genes necessary for egg production in flies.

Different strategies can be used to determine whether the SNP in the predicted PRE allows *rib* expression in the ovaries to rescue the fertility defect caused by null *BEAF* mutations. One approach would be to use fluorescence in situ hybridization (FISH) to screen for atypical *rib* expression in *Tofu* ovaries. Another approach would be to use the GAL4 *UAS* system with different GAL4 drivers to determine if *UAS::rib* expression in different ovary cell types can rescue the *ABKO* mutation. Another possibility would be to use CRISPR-Cas9 to introduce or correct the PRE mutation and determine the effect on fertility in the presence of a null *BEAF* mutation. If it is found that *Tofu* is not a *rib* ovary misexpression allele and the PRE SNP is not involved, the genome sequences that we have generated provide a valuable resource for identifying other candidates. This could start with a deeper analysis of the SNPs in REDfly CREs.

## Figures and Tables

**Table 1:** Genomic sequencing statistics.

Sample	Number of Reads per End	Filtered, Clipped, Paired, and Mapped Reads	Mean Quality (PHRED)	Mean Fragment (bp)	Fold Genome Coverage
ABKO	32,067,905	19,177,162	33	347	48
Tofu KO	29,721,768	16,853,622	36	363	42
NP6377	31,616,665	19,035,482	36	361	47
Tofu NP	28,497,357	16,010,932	36	372	40
y^-^ w^-^	28,514,732	16,028,674	36	355	40

**Table 2:** Chromosome arm sizes and SNP counts per chromosome in sequenced lines.

	chr2L	chr2R	chr3L	chr3R	chrX	chr4	Total
Size (Mb)	23.51	25.29	28.11	32.08	23.54	1.35	133.88
NP6377	164,335	148,240	181,649	149,138	121,174	2159	766,695
Tofu NP	215,087	192,164	192,112	179,544	113,948	1872	894,727
Tofu KO	168,156	145,573	191,076	179,089	106,302	1373	791,569
ABKO	166,805	145,117	229,148	186,378	119,324	1400	848,172
y^-^ w^-^	167,007	148,868	200,959	150,514	104,656	1156	773,160
Tofu Pattern	71	1132	34,711	20,940	4783	56	61,693

Tofu pattern: SNPs present in the *NP6377*, *Tofu NP*, and *Tofu KO* sequences but absent in the *ABKO* and *y- w-* sequences.

## Data Availability

The raw WGS genomic sequencing files are available at https://www.ncbi.nlm.nih.gov/bioproject/?term=PRJNA1069327 (accessed on 24 February 2026). Other data presented in this study are included in the article and Supplementary Materials. Further inquiries can be directed to the corresponding author.

## Supplementary Materials

The following supporting information can be downloaded at https://www.mdpi.com/article/10.3390/genes17030328/s1. File S1. fileS1_np63dm6.raw.snps.indels.vcf: VCF file for the original *BEAF^NP6377^* fly stock with *Tofu* and at least one recessive lethal mutation; File S2. fileS2_npnpdm6.raw.snps.indels.vcf: VCF file for the fly stock with *BEAF^NP6377^* and *Tofu* but lacking any recessive lethal mutation; File S3. fileS3_kokodm6.raw.snps.indels.vcf: VCF for the fly stock with the *BEAF^AB-KO^* and *Tofu* alleles; File S4. fileS4_kog30dm6.raw.snps.indels.vcf: VCF for the original fly stock with *BEAF^AB-KO^* but lacking *Tofu*; File S5. fileS5_whitedm6.raw.snps.indels.vcf: VCF for the *y^-^ w^-^* fly stock used as a wild-type genome; File S6. fileS6_tofu.vcf: VCF filtered to only retain variants unique to fly stocks with *Tofu* (present in *NP6377*, *Tofu NP*, and *Tofu KO* but not in *ABKO* or *y^-^ w^-^*); File S7. fileS7_tofuchrom2.vcf: VCF with snpEff analysis of chr2 variants unique to fly stocks with *Tofu* (present in *NP6377*, *Tofu NP*, and *Tofu KO* but not in *ABKO* or *y^-^ w^-^*); Supplementary Tables S1–S3: Table S1: Excel table summarizing the SnpEff evaluation of chromosome 2 SNPs; Table S2: Excel table of SNPs matched to REDfly CREs; Table S3: Excel table of SNPs matched to PREs.
